# Genome Sequence of the Neurotoxigenic *Clostridium butyricum* Strain 5521

**DOI:** 10.1128/genomeA.00632-14

**Published:** 2014-06-26

**Authors:** Karl A. Hassan, Liam D. H. Elbourne, Sasha G. Tetu, Eric A. Johnson, Ian T. Paulsen

**Affiliations:** a Department of Chemistry and Biomolecular Sciences, Macquarie University, NSW, Australia; b Department of Bacteriology, Botulinum Toxins Laboratory, Food Research Institute, Madison, Wisconsin, USA; c J. Craig Venter Institute, Rockville, Maryland, USA

## Abstract

*Clostridium* strains from six phylogenetic groups, *C. botulinum* groups I to IV, *C. baratii*, and *C. butyricum*, display the capacity to produce botulinum neurotoxin. Here, we present the genome sequence of a *C. butyricum* isolate, the neurotoxigenic strain 5521, which encodes the type E botulinum neurotoxin.

## GENOME ANNOUNCEMENT

*Clostridium butyricum* is a Gram-positive bacillus named for its capacity to produce butyric acid. *C. butyricum* strains are found in a variety of environments and are common human and animal gut commensals. However, some *C. butyricum* strains have been found to cause the paralytic condition botulism (1–4). These neurotoxigenic *C. butyricum* strains harbor an operon encoding the type E botulinum neurotoxin (BoNT/E) likely to have been horizontally acquired (5). Neurotoxigenic *C. butyricum* strains were originally identified as the cause of botulism in infants in Italy (1, 2) and have since been identified in other countries. Here, we present the genome sequence of a neurotoxigenic *C. butyricum* strain, 5521, isolated from a case of botulism. The genome sequence of *C. butyricum* 5521 was determined to 8-fold coverage using Sanger shotgun sequencing. The sequence reads were assembled and annotated as previously described (6). The sequence is organized into 123 scaffolds and includes 3,827 putative protein-coding genes and 259 predicted pseudogenes.

The predicted protein-coding potential of *C. butyricum* 5521 is similar to that of *C. butyricum* BL 5262 (5); tblastx 2.2.5+ searches demonstrated that >96% of the 4,086 genes or pseudogenes of *C. butyricum* 5521 are shared between these two strains (*E* value ≤ 1e^-30^). Of the clostridial species for which complete genome sequences are available, 16S rRNA analyses have suggested that *C. butyricum* is most closely related to group II *Clostridium botulinum*, *Clostridium acetobutylicum*, and *Clostridium beijerinckii* (3). tblastx comparisons indicate that 2,352, 1,809, and 2,568 genes or pseudogenes of *C. butyricum* 5521 have orthologs within the genomes of *C. botulinum* E3 strain Alaska E43 (5) (Genbank accession no CP001078), *C. acetobutylicum* ATCC 824 (7), and *C. beijerinckii* NCIMB 8052 (8), respectively.

A defining characteristic of *C. butyricum* strains is their production of butyric acid. The enzymes involved in butyric acid production from acetyl-coenzyme A (CoA) in *C. butyricum* 5521 are encoded primarily within two gene clusters, CBY_2919-20 and CBY_3041-45 (both on scaffold ABDT01000094 in the current assembly). However, acetyl-CoA acetyltransferase, CBY_1290, which mediates the first step in this pathway (9), is encoded elsewhere (scaffold ABDT01000114).

The gene encoding the BoNT/E toxin within *C. butyricum* 5521 is present in a gene cluster localized within the chromosomal *rarA* resolvase locus. This gene cluster is identical to that carried by the neurotoxigenic *C. butyricum* strain BL 5262, which is also localized within the *rarA* locus (5). A detailed analysis of this gene cluster demonstrated that it shows an organization that is highly similar or identical to that of the BoNT/E gene clusters carried by group II type E toxin-producing *C. botulinum* strains (5).

The genome sequence of *C. butyricum* 5521 will prove useful for comparative studies and for future investigations of type E botulism.

### Nucleotide sequence accession numbers.

The draft genome sequence for *C. butyricum* 5521 has been added to the GenBank database under the accession no. ABDT00000000. The version described here is ABDT00000000.1.
